# Ethical decision-making climate, moral distress, and intention to leave among ICU professionals in a tertiary academic hospital center

**DOI:** 10.1186/s12910-022-00775-y

**Published:** 2022-04-19

**Authors:** Henry Silverman, Tracey Wilson, Samuel Tisherman, Raya Kheirbek, Trishna Mukherjee, Ali Tabatabai, Karen McQuillan, Rachel Hausladen, Melissa Davis-Gilbert, Eunsung Cho, Kerri Bouchard, Samantha Dove, Julie Landon, Michele Zimmer

**Affiliations:** 1 University of Maryland School of Medicine, Baltimore, MD 21201 USA; 2 Research Analytics and Design, SCorp, New York, USA; 3 University of Maryland Medical Center, Baltimore, USA

**Keywords:** Ethical climate, Moral distress, Intention to leave, Nurses, Physicians, Decision making for end-of-life care, Interprofessional collaboration

## Abstract

**Background:**

Commentators believe that the ethical decision-making climate is instrumental in enhancing interprofessional collaboration in intensive care units (ICUs). Our aim was twofold: (1) to determine the perception of the ethical climate, levels of moral distress, and intention to leave one's job among nurses and physicians, and between the different ICU types and (2) determine the association between the ethical climate, moral distress, and intention to leave.

**Methods:**

We performed a cross-sectional questionnaire study between May 2021 and August 2021 involving 206 nurses and physicians in a large urban academic hospital. We used the validated Ethical Decision-Making Climate Questionnaire (EDMCQ) and the Measure of Moral Distress for Healthcare Professionals (MMD-HP) tools and asked respondents their intention to leave their jobs. We also made comparisons between the different ICU types. We used Pearson's correlation coefficient to identify statistically significant associations between the Ethical Climate, Moral Distress, and Intention to Leave.

**Results:**

Nurses perceived the ethical climate for decision-making as less favorable than physicians (*p* < 0.05). They also had significantly greater levels of moral distress and higher intention to leave their job rates than physicians. Regarding the ICU types, the Neonatal/Pediatric unit had a significantly higher overall ethical climate score than the Medical and Surgical units (3.54 ± 0.66 vs. 3.43 ± 0.81 vs. 3.30 ± 0.69; respectively; both *p* ≤ 0.05) and also demonstrated lower moral distress scores (both *p* < 0.05) and lower “intention to leave” scores compared with both the Medical and Surgical units. The ethical climate and moral distress scores were negatively correlated (r = −0.58, *p* < 0.001); moral distress and "intention to leave" was positively correlated (r = 0.52, *p* < 0.001); and ethical climate and “intention to leave” were negatively correlated (r = −0.50, *p* < 0.001).

**Conclusions:**

Significant differences exist in the perception of the ethical climate, levels of moral distress, and intention to leave between nurses and physicians and between the different ICU types. Inspecting the individual factors of the ethical climate and moral distress tools can help hospital leadership target organizational factors that improve interprofessional collaboration, lessening moral distress, decreasing turnover, and improved patient care.

**Supplementary Information:**

The online version contains supplementary material available at 10.1186/s12910-022-00775-y.

## Introduction

During the last few decades, the rapid technological advancements occurring in intensive care units (ICU) present significant ethical challenges with decision-making for clinicians caring for critically ill patients at the end-of-life. An ethically-based climate for decision-making promotes interprofessional collaboration and mutual respect, a culture of ethical awareness, and authentic leadership by physicians guided by the values of patients and their family members. Such a climate is an essential component of ICU functioning [1]. An ethical decision-making climate has been defined as: "A climate in which clinicians are empowered to speak up and in which they feel that their opinion is valued and subsequently integrated into the decision-making process" [2].

Studies have indicated that interprofessional collaboration is lacking, and consequently, the ICU decision-making process for end-of-life decision-making has not been well managed [1–3]. A poor ethical climate in which healthcare team members are not empowered to participate in the decision-making process or advocate for the patient can engender interdisciplinary and family-staff conflicts, leading to excessive treatments, many of which are considered medically futile. The result includes patients with reduced and often unmanageable quality of life near death and many professional staff experiencing moral distress.

Jameton initially defined moral distress as the inability to act according to an individual’s ethical beliefs due to structural or hierarchical constraints prevalent in the ICUs [4]. This pressure to act unethically is the defining concept of this phenomenon that can threaten moral integrity and differs from situations that are emotionally distressing or morally troubling (e.g., acting under uncertainty in the presence of an ethical dilemma). While the nursing profession has recognized and investigated the importance of moral distress for the last few decades, awareness of moral distress within physician groups has only been recently recognized [5, 6]. Many instruments have been developed to measure moral distress. The recent Measure of Moral Distress for Healthcare Professionals (MMD-HP) includes additional root causes of moral distress, and several investigators have used the instrument with nurses, physicians, and other professionals [3, 7]. The key components of the MMD-HP account for three levels of root causes of moral distress (patient, unit, and system) that include issues related to interprofessional collaboration regarding decision making. Examples of these issues include “lack of a voice," “poor team communication," “feel pressures to participate in care that one does not agree with," and lack of team communication.” [7]

Studies in health care settings have identified that the more positive the ethical climate is perceived, the greater is the interprofessional collaboration that leads to lower levels of moral distress [3, 7, 8]. Recent evidence indicates that the quality of the ethical climate and levels of moral distress is associated with leaving one's job [9–12]. Clinician burnout is linked to poor clinician well-being, job dissatisfaction, and job turnover [13].

Recently, Van den Bulcke and colleagues reported on developing and validating an Ethical Decision-Making Climate Questionnaire (EDMCQ) [1]. This tool allows one to measure certain domains of the ethical climate that reflect interprofessional collaboration, values-based reflections and discussions, mutual respect, and empowering leadership by physicians. The EDMCQ was used in 68 ICUs in 13 European countries and the United States [2] and the results showed that physicians consistently perceived the ethical decision-making climate more favorably than nurses. The most significant differences between physicians and nurses occurred in the ethical climate domains regarding “physician leadership," "interdisciplinary reflection," and “a culture of not avoiding end-of-life decisions." Other studies have investigated the hospital’s ethical climate that used the EDMCQ [3].

However, fewer studies have explored the association of ethical climate, moral distress, and intention to leave one's job [8, 9]. Our goal was to add to the current literature investigating the relationship between these three constructs in a large urban medical academic health center. Our aims were two-fold: (1) to investigate variations in these three constructs between nurses and physicians and between the different types of ICUs: medical, surgical, and neonatal/pediatrics, and (2) to explore the associations between the perceptions of the ethical climate, levels of moral distress, and the intention to leave one’s job.

## Methods

### Study design

We performed a cross-sectional questionnaire study.

### Study time-period

We conducted our study between May 2021 and August 2021. Baltimore had witnessed the second peak of Covid-19 in mid-January 2021 with approximately 1950 hospitalizations that decreased by mid-March, but then April saw hospitalizations climb back up to over 1,000 patients before dropping off significantly over the summer [14].

### Setting

A tertiary academic university that included the following ICUs: Medical ICUs (medical intensive care unit, coronary care unit, and intermediate medical care unit), Surgical ICUs (surgical, cardiothoracic, neurotrauma, neurotrauma, trauma, and lung rescue unit), and Neonatal/Pediatric ICUs.

### Participants

Participating staff included nurses (bedside nurses, nurse managers, and certified registered nurse practitioners) and physicians (attending intensivists, ICU consultants, fellows, and residents).

### Recruitment

We asked ICU directors and nurse managers to send emails to their respective staff informing them of a survey involving the ethical decision-making climate of the ICU, the experience of moral distress, and job satisfaction. Subsequently, in each ICU, flyers and posters were displayed that provided information about the purpose of the study and a QR code that contained a link to the questionnaire on www.Surveymonkey.com. While leadership in the ICUs informed the staff about the survey, they had no substantive role in recruitment that would affect selection.

After accessing the link, participants would be presented with an information page that provided more details of the survey study. Participants indicated their informed consent for participation if they clicked on the “continue” button. Responses were collected anonymously. Data were securely stored on the www.surveymonkey.com platform, and after enrollment was completed, we downloaded the data on our password-protected computers. None of the responses could be traced back to the participants. Participation was strictly voluntary.

We offered an incentive to increase enrollment from individuals who might not be interested in the study topic or feel they might not have the time. Participants who completed the survey could choose to enter a lottery to claim an Amazon $50 gift card. This lottery occurred after every 50 participants completed the survey. After enrollment ended, we conducted another lottery that offered an Apple iPad. Participants indicated their preference to participate in the lottery by sending an email several days after completing the questionnaire to the principal investigator. The time delay in sending an email represented a technique to assure privacy. The average time to complete the survey was 19 min and 11 s.

### Survey tools

#### Ethical climate tool

We used the self-assessment Ethical Decision-Making Climate Questionnaire (EDMCQ) developed and validated by Van den Bulcke [1]. The questionnaire consists of 32 items with 5-point Likert scale options to indicate a level of agreement (strongly disagree to strongly agree) or frequency of occurrence (never, seldom, occasionally, often, and always). The exploratory factor analysis performed by Van den Bulcke and colleagues showed that the EDMCQ consists of seven distinct ethical climate factors: F1: self-reflective and empowering leadership by physicians; F2: practice and culture of open interdisciplinary reflection and communication; F3: culture of not avoiding end-of-life decisions; F4: culture of mutual respect within the interdisciplinary team; F5: active involvement of nurses in end-of-life care and decision-making; F6: active decision-making by physicians; and F7: Practice and culture of ethical awareness and support. Additional file 1 shows the individual question items within each factor.

To identify distinct ethical climates within our sample size, we explored dimension reduction through cluster analysis using the identified seven factors (Benoit, et al. [2]). Each unit within the ICU consists of nurses and physicians who have their perception of the ethical climate. The individual responses were converted into scores between 1 and 5 (Strongly Disagree—Strongly Agree). The average score across health care providers across the different factors for different ICU professionals was calculated. The mean (± SD) scores across various ethical climate factors were compared between the other ICU staff and ICU types. At the same time, significant differences were identified through Games-Howell post-hoc analysis (*p*-value ≤ 0.05). The Games Howell post-hoc test assumes homogeneity of variances. The Levene’s test was used on ICU staff and ICU types to test for homogeneity of variances (*p*-value < 0.001).

Similar to Benoit et al., during dimensional reduction, we performed varimax rotation [2]. The average score (between 1 and 5) was used as input for the cluster analysis at the ICU level. The responses were subsequently clustered into ethical climates using the partitioning around medoids (PAM) algorithm, which seeks to minimize the similarity of responses within each cluster and maximize the dissimilarity of responses between clusters.

We used silhouette analysis to determine the ideal number of clusters, which measures how well an observation is clustered and estimates the average distance between clusters. We used Model-based clustering based on parameterized finite Gaussian mixture models for clustering. Models were estimated by the Expectation–Maximization algorithm initialized by hierarchical model-based agglomerative clustering. The optimal model was then selected according to the Bayesian Information Criterion (BIC).

We found scores to cluster within four mutually exclusive ethical climates. Our cluster analysis is shown in Additional file 2. The mean scores (SD) and the internal consistency of the identified seven climate factors are shown in Additional file 3.

#### The measure of moral distress for healthcare professionals tool (MMD-HP)

The MMD-HP consists of 27 different clinical situations and an option to suggest other clinical situations of moral distress [7]. Participants rated each item on a Likert scale for how often it occurs in their practice (frequency: 0 = never to 4 = very frequently) and for how distressing it is when it happens (distress: 0 = none to 4 = very distressing).

We performed an exploratory factor analysis (EFA) to identify the underlying factors between item scores in the MMD-HP and thus to check the construct validity of the scales. The number of factors was determined using the Kaiser Criterion and the scree plot. A factor loading cut-off value of 0.30 was chosen to decide which items were highly associated within a given factor; as such, only items correlating 0.30 or higher with a factor in the rotated solution were considered.

The moral distress score of each item in the MMD-HP was calculated by multiplying the frequency score of occurrences (f) by the level of distress score (d) to create a *composite score* (range 0–16). Each factor's total moral distress score was calculated by summing the *composite scores* of the items associated with the factor and dividing this sum by the number of items per factor. To compare moral distress scores between professional staff and ICU types, we used Analysis of Variance (ANOVA) with the Games-Howell post-hoc test to correct multiple testing. We also testing for homogeneity of variances using Levene’s test (*p*-value < 0.001). The factors were interpreted by examining the pattern matrix.

The EFA of the MMD-HP identified four different factors, which explained 57% of the variance. Additional dile 4 shows the identified four factors and the associated factor loadings for each item of the Measure of Moral Distress-Healthcare Professionals (MMD-HP) tool. Three question items from the original MMD-HP were eliminated due to low factor loadings. Additional file 5 shows that each factor of the MMD-HP demonstrated good to acceptable internal consistency as the Cronbach’s alphas ranged from 0.77 to 0.85**.** The descriptions of the individual factors were interpreted by examining the pattern matrix.

### Intention to leave

Clinicians were asked to report whether they actively considered leaving their current job [12]. The specific question asked was: "I have thoughts about leaving my current position/job." Results were converted from Likert responses ranging from 1–5 (Strongly Disagree = 1; Strongly Agree = 5). We used Games-Howell post-hoc analysis (*p*-value ≤ 0.05) to compare mean “intention to leave scores” within the other professional staff and between the different ICU types.

### Correlation analysis

Pearson’s correlation coefficient was computed to identify statistically significant associations between moral distress and ethical climate, moral distress, and intention to leave, and ethical climate and intention to leave. RStudio v1.2.5 was used for all statistical and descriptive analyses.

### Assumption testing

Pearson’s correlation coefficient assumes linearity which was tested by inspecting the distribution of residual errors in the data for moral distress and intention to leave (Panel A Additional file 6) and ethical climate and intention to leave (Panel B Additional file 6).

### Ethics

This study was approved by the Institutional Review Board at the University of Maryland Baltimore, #HP-00095943.

## Results

Of the 206 participants enrolled in the survey study, nurses represented almost 73% of the participants, while physicians comprised slightly more than a quarter (27%). This proportion between nurses and physicians compares with other studies using the EDMCQ tool [3, 15]. The Medical and Surgical ICUs contributed similar proportions of participants (42.7% and 40.2%, respectively), while the Neonatal/Pediatric Units contributed 17.1%. (For further details, see Table 1). *Assumption testing-Test for homogeneity of variances:* The *p*-value for Levene’s test across all our tested parameters was < 0.001 indicating that the assumption of homogeneity is lacking.

**Table 1:** Participant demographics (n = 206)

	Number	Percent (%)
Gender		
Female	151	73.3
Male	52	25.2
Other	3	1.5
Role within ICU		
Nurse	151	73.3
Physician	55	26.7
Participants within each ICU type		
Medical—total	88	42.7
Surgical—total	83	40.2
Neonatal/pediatric	35	17.1

### Ethical climate scores: nurses and physicians

#### Overall ethical climate scores

The nurses' overall mean ethical climate score was significantly lower than for physicians (3.30 ± 0.69 vs. 3.69 ± 0.65; respectively (*p* ≤ 0.05) (See Table 2).

**Table 2:** Ethical climate scores per professional staff and ICU types

Role within ICU	Mean (S.D.)^a^		*p*-value
	Physician	Nurses	P-N^b^
Overall ethical climate	3.69 (0.65)	3.30 (0.69)	< 0.05
Factors			
1. Self-reflective and empowering leadership by physicians	3.48 (0.81)	3.31 (0.90)	< 0.05
2. Practice and culture of open interdisciplinary reflection	3.7 0(0.75)	3.60 (0.79)	0.4
3. Culture of not avoiding EOL-DM	2.9 0(0.82)	2.40 (0.83)	< 0.05
4. Mutual respect within the interdisciplinary team	4.20 (0.59)	3.66 (0.84)	< 0.001
5. Active involvement of nurses in EOL care and decision-making	3.43 (0.73)	3.50 (0.72)	0.2
6. Active Decision-Making by physicians	3.69 (0.79)	3.01 (0.89)	0.03
7. Practice and culture of ethical awareness	3.70 (0.70)	3.20 (0.85)	0.03

ICU types	Mean (S.D.)^a^			*p*-value		
	Medical ICUs	Neonatal/pediatric	Surgical ICUs	NP-M^c^	M-S^d^	NP-S^e^
Overall ethical climate	3.43 (0.81)	3.54 (0.66)	3.30 (0.69)	.05	.03	< 0.001
Factors						
1. Self-reflective and empowering leadership by physicians	3.35 (0.75)	3.47 (0.47)	3.33 (0.6)	0.15	0.45	0.1
2. Practice and culture of open interdisciplinary reflection	3.63 (0.60)	3.60 (0.73)	3.64 (0.59)	0.42	0.45	0.40
3. Culture of not avoiding EOL-DM	2.68 (0.81)	2.87 (0.90)	2.39 (0.92)	0.16	0.01	0.01
4. Culture of mutual respect within the interdisciplinary team	3.82 (0.72)	4 (0.77)	3.69 (0.70)	0.1	0.2	0.01
5. Active involvement of nurses in EOL care and decision-making	3.47 (0.93)	3.47 (0.80)	3.25 (0.96)	0.11	0.12	0.05
6. Active Decision-Making by physicians	3.4 (0.77)	3.6 (0.73)	3.54 0.68)	0.07	0.08	0.2
7. Practice and culture of ethical awareness	3.73 (0.68)	3.63 (0.56)	3.25 (0.82)	0.3	< 0.001	< 0.001

^a^Score range of 1–5 higher scores reflect a better perceived ethical climate

^b^Physicians versus nurses

^c^Neonatal/pediatric versus medical

^d^Medical versus surgical

^e^Neonatal/pediatric/surgical

#### Individual ethical climate factors

The EDMCQ tool incorporates seven distinct ethical climate factors. Additional file 1 shows the individual question items within each factor.

Compared with nurses, physicians demonstrated significantly higher ethical climate scores on all factors except for: “Practice and culture of open interdisciplinary reflection” and “Active involvement of nurses in end-of-life care and decision-making.” The climate factor “Not avoiding end-of-life decisions” was the lowest ethical climate factor for both nurses and physicians. However, physicians demonstrated a significantly higher mean score on this factor than nurses (2.9 ± 0.82 vs. 2.4 ± 0.83 vs.; respectively, *p* = 0.05) (For further details, see Table 2).

### Ethical climate scores: distinct ICUs

#### Overall ethical climate scores

The Neonatal/Pediatric units had a significantly higher overall ethical climate score (3.54 ± 0.66) compared with the Medical ICUs (3.43 ± 0.81; *p* = 0.05) and Surgical ICUs (3.30 ± 0.69; *p* < 0.001). The Medical ICUs had a significantly higher overall climate score than the Surgical ICUs; see Table 3.

**Table 3:** Percentage professional staff and ICU types within the individual ethical climates

	Good (%)	Average (+) (%)	Average (−) (%)	Poor (%)
*Professional staff*				
Physicians (n = 55)	29.1	34.5	25.5	10.9
Nurses (n = 151)	15.9	23.2	29.8	31.1
*ICU types*				
Medical (n = 88)	18.2	25	30.7	26.1
Surgical (n = 83)	16.9	22.9	28.9	31.3
Neonatal/Pediatric (n = 35)	28.6	37.1	22.9	11.4
Total	19.4	26.2	28.6	25.7

A higher percentage of physicians perceived their ethical climate as "good" than the nurses. A higher percentage of the professional staff in the Neonatal/Pediatric ICUs rated their ethical climate “good” compared with the Medical and Surgical ICUs

#### Individual ethical climate factors

There were no significant differences for any of the climate factors between the Neonatal/Pediatric ICUs and the Medical ICUs. In contrast, the Neonatal/Pediatric ICUs and the Medical ICUs had significantly higher climate scores for the factors “Culture of not avoiding end-of-life decision-making” and “Practice of culture and awareness” compared with the Surgical ICUs. Additionally, the Neonatal/Pediatric ICUs compared with the Surgical ICUs had significantly higher climate factor scores for “Culture of mutual respect within the interdisciplinary team” and “Active involvement of nurses in end-of-life care and decision making”. These two climate factors are pivotal for enhancing interprofessional collaborations. The climate factor “Culture of not avoiding end-of-life decisions” factor received the lowest scores for all ICU types (see Table 2 for further details).

### Distinct ethical climate types

**Fig. 1:**
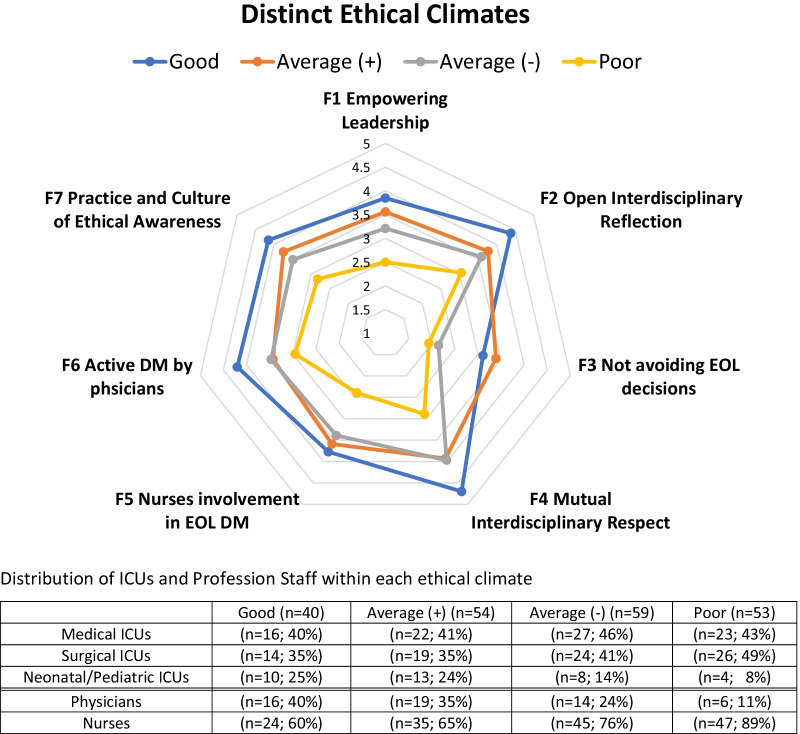
Cluster analysis identified four meaningful ethical climates: good, average (+), average (−) and poor. The figure visualizes the mean scores of each factor per climate. Larger values of each factor indicate a more positive environment for decision making

Similar to the analysis of Benoit and colleagues, our cluster analysis based on the average scores of the seven identified factors [2] yielded four different meaningful, mutually exclusive ethical climates. Using the previous terminology of Benoit and colleagues, we characterized these climates as "good"; "average (+)," "average (−)” and “poor." Fig. 1 shows a visual representation of these distinct climate types.

The”good” climate is distinguished from the “average (+)” climate by having considerably higher scores on all of ethical climate factors except “F3: not avoiding end-of-life decisions”. The greatest differences between these two climates occurred with F2: “open interdisciplinary reflection”; F4: “mutual interdisciplinary respect”; F6: “active decision making by physicians”; and F7: “Practice and culture of ethical awareness”. The “average (+)” climate differs from the “average (−)” climate by having higher scores for five of the seven climate factors. The climate factor with the greatest discordance in the scores between these climates was “Not avoiding end-of-life decision making. The “poor” climate had exceptionally lower scores on all climate factors compared with the other ethical climates. Further details regarding the mean scores of each climate type are shown in Additional file 7.

#### Percentage of staff and ICUs among the different ethical climates

Approximately a quarter of the professional staff perceived their ethical climate as being “average (+)”, “average (−)”, or “poor”. A smaller percentage (19.4%) rated their ethical climate as “good”.

Regarding individual professional types, a smaller percentage of nurses (15.9%) perceived their ethical climate as "good" (15.9%) compared with physicians (29.1%) Approximately a quarter of the nurses and physicians rated their ethical climate as average (−). A higher proportion of nurses (31.1%) perceived their ethical climate as poor compared to physicians (10.9%).

A higher percentage of the professional staff in the Neonatal/Pediatric ICUs rated their ethical climate as “good” (28.6%) compared with the Medical and Surgical ICUs (18.2% and 16.9%; respectively). A larger percentage of the staff in the Surgical ICUs, rated their ethical climate as “poor” compared with the other units. See Table 3 for further details.

### The measure of moral distress in healthcare professionals (MMD-HP)

The Exploratory Factor Analysis of the MMD-HP revealed four distinct factors, see Table 4. Factor 1 represents patient-level root causes of moral distress due to suboptimal decision-making. Factor 2 represents organizational-level root causes of moral distress due to organizational restrictions and burden. Factors 3 and 4 represent team-level root causes of moral distress. Factor 3 involves suboptimal patient care due to inadequate communications and professionalism integrity, and Factor 4 represents a culture of fear and lack of empowerment. The items associated with each factor is shown in Additional file 4.

**Table 4:** Mean moral distress scores per professional staff and ICU Types

	Mean (S.D.)^a^		*p*-value
Role within ICU	Physician	Nurse	P-N^b^
Overall moral distress	4.27 (3.0)	4.67 (2.9)	0.04
Factors			
1. Ethically Inappropriate Care Due to Suboptimal Clinical Decision Making	6.66 (2.71)	7.6 (2.49)	0.03
2. Suboptimal patient care due to organizational restrictions / burden	4.32 (2.13)	4.7 (2.49)	0.14
3. Suboptimal quality of care due to poor team communication or lack of professionalism	4.17 (2.44)	4.5 (2.31)	0.03
4. Culture of fear and power hierarchy	2.18 (2.99)	2.05 (2.84)	0.82

	Mean (S.D.)^a^			*p*-value		
ICU types	Medical	Pediatric	Surgical	NP-M^c^	M-S^d^	NP-S^e^
Overall moral distress	4.57 (2.7)	4.05 (2.8)	4.54 (2.9)	0.02	0.85	0.01
Factors						
1. Ethically inappropriate care due to suboptimal clinical decision making	7.35 (2.7)	6.84 (2.9)	6.98 (2.2)	0.27	0.23	0.73
2. Suboptimal patient care due to organizational restrictions / burden	4.43 (2.9)	3.39 (2.9)	4.18 (1.7)	0.002	0.32	0.01
3. Suboptimal quality of care due to poor team communication or lack of professionalism	3.72 (2.7)	3.88 (2.6)	4.33 (1.7)	0.76	0.12	0.38
4. Culture of fear and power hierarchy	2.00 (2.6)	2.00 (2.0)	2.39 (1.9)	0.10	0.14	0.20

^a^Score range 0–16 higher scores reflect higher moral distress scores

^b^Physicians versus nurses

^c^Neonatal/pediatric versus medical

^d^Medical versus Surgical

^e^Neonatal/pediatric/surgical

### Moral distress scores

#### Overall moral distress scores per profession

Nurses had significantly higher overall moral distress scores than physicians (4.67 ± 2.9 vs. 4.27 ± 2.95, respectively, *p* = 0.04).

#### Moral distress scores per factor for each profession

The factors “Ethically Inappropriate Care Due to Suboptimal Clinical Decision Making” and “Suboptimal quality of care due to poor team communication or lack of professionalism” represented higher moral distress for nurses compared with physicians (see Table 4).

#### Overall moral distress levels per ICU type

The professional staff in the Neonatal/Pediatric units experienced less moral distress than the Surgical and Medical ICUs. Moral distress scores were similar between the Surgical and Medical ICUs (See Table 4).

#### Moral distress scores per factor for each ICU type

Moral distress scores for “Ethically Inappropriate Care Due to Suboptimal Clinical Decision Making” and “Culture of fear and power hierarchy” were significantly higher for the Adult ICUs compared with the Neonatal/Pediatric ICUs (See Table 4).

### Intention to leave

Nurses had higher "intention to leave" scores compared with physicians. The Neonatal/Pediatric ICUs had significantly lower "intention to leave” score than the Medical and ICUs. There were no significant different in the “intention to leave” score between the Adult ICUs (See Table 5).

**Table 5:** Mean “intention to leave” scores among the different professional types and the different ICU Types

	Mean score (S.D.)^a^		*p*-value
Role within ICU	Physician	Nurse	P-N^b^
Intention to leave	2.85 (1.3)	3.24 (1.3)	0.04*

ICU grouping	Medical	Neonatal/Pediatric	Surgical	NP-M^c^	M-S^d^	NP-S^e^
Intention to leave	3.21 (1.3)	2.87 (1.3)	3.15 (1.4)	0.04*	0.8	0.07

^a^Score range 1–5 higher scores reflect greater intention to leave

^b^Physicians versus nurses

^c^Neonatal/pediatric versus medical

^d^Medical versus surgical

^e^Neonatal/pediatric/surgical

The percentage of nurses’ responses regarding their “intention to leave” (combined agree/strongly agree) were great than those of physicians (54% vs. 38%, respectively). The “intention to leave” responses (combined agree/strongly agree) for the Medical, Surgical, and Neonatal/Pediatrics ICUs were similar (50%, 48%, and 44%; respectively).

### Correlation between ethical climate, moral distress, and intention to leave

**Fig. 2:**
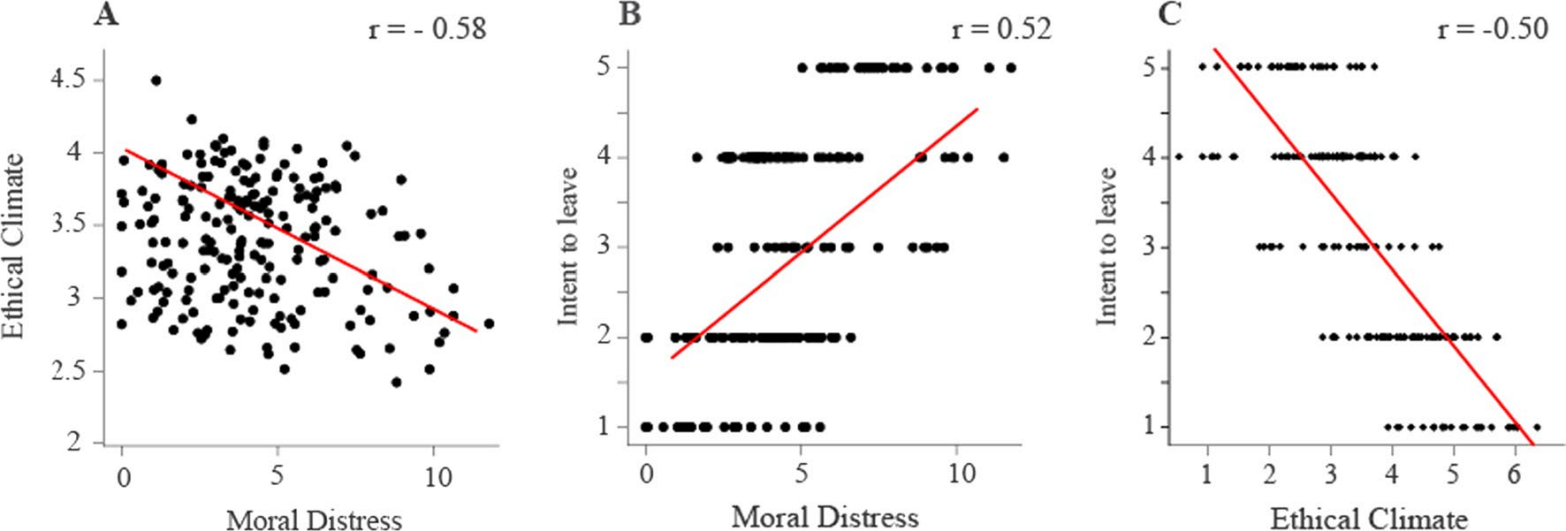
Correlation analysis between measures of ethical climate and moral distress (**A**), Intention to leave and moral distress (**B**), and Intention to Leave and ethical climate (**C**)

We found a lack of pattern in the plot of residuals and fitted data for moral distress and intention to leave and ethical climate and intention to leave indicating linearity in the data (see Additional file 6). Moral distress and ethical climate were found to be negatively correlated (r = −0.58, *p* < 0.001) across all professional types (Fig. 2A); moral distress and “intention to leave” was positively correlated (r = 0.52, *p* < 0.001) (Fig. 2B) and ethical climate and intention to leave were negatively correlated (r = −0.50, *p* < 0.001) (Fig. 2C).

## Discussion

Our study showed that in a large urban academic center, the “intention to leave” was correlated with levels of moral distress and inversely correlated with the perception of the ethical climate for decision-making as measured by the EDMCQ tool. These results suggest that a deficient ethical climate for decision-making plays a significant role in developing moral distress in health care providers, which represents an influential factor for their “intention to leave” their jobs. These findings harbor considerable importance for a significant segment of our healthcare service sector, as nurses perceived the ethical climate less favorably than physicians, demonstrated higher levels of moral distress, and had a greater intention to leave compared with physicians. Dzeng and Curtis proposed a conceptual framework that emphasizes a causal pathway from the ethical climate to the intensity of end-of-life care with subsequent effects on clinicians’ moral distress and the intention to leave [16].

### Intention to leave

Our results corroborate the findings of other studies showing that the ethical climate has an inverse association between levels of moral distress and the intention to leave one’s job [8–12]. For example, in a multicenter study of physicians and nurses in adult ICUs, investigators demonstrated an inverse association between the EDMCQ tool and the intention to leave [12]. Hamric and colleagues found that nurses with higher moral distress scores were more likely to have left or considered leaving a position than nurses with lower scores [13]. Their study also showed that nurses perceived their ethical environment as more negative, experienced higher moral distress scores, and rated their collaboration within their teams less favorably than physicians.

Job turnover, preceded by clinician burnout, can adversely affect patient care, including reduced patient satisfaction, quality of care, patient rapport, and patient safety with higher rates of medical errors and declines in empathy [17, 18]. High levels of moral distress and burnout impact staff health, well-being, performance, patient safety, experience, and quality of care [19–23]. Others have recognized the influence of the ethical climate in determining well-being of health professionals [1, 16, 23–25].

The percentage of nurses who expressed an intention to leave was 54%, which contrasts with other studies reporting lower rates of an intention to leave or “burnout. For example, van den Bulcke and colleagues found that in 68 ICUs across Europe and the United States during March–May 2014, nurses' average intention to leave was 27.0% [12]. In a study performed in ICUs in Sao Paulo, Brazil during August to September 2015, the burnout rate of ICU nurses was 28.6% [26]. Hamric and Blackhall reported in 2007 that 28% of ICU nurses expressed an intention to leave [13].

However, the higher prevalence of an intention to leave we demonstrated could be explained by our survey occurring after the 2nd/3rd Covid-19 peak. To illustrate, in a recent interview study involving nurses in the same hospital system, we identified a wide range of causes for nurses’ moral distress that were specific for caring for patients with Covid-19. These included increased workload from higher patient/nurse ratios, greater disagreements regarding appropriate use of end-of-life treatments, not having their voices “heard” in plan of care decisions, and frustration with not assuming the traditional care ethics of nursing [27]. This premise regarding higher levels intention to leave scores receives support from the study conducted by Chor and colleagues who surveyed emergency department nurses and physicians in Singapore during the Covid pandemic and found that a significant proportion of respondents reported high moderate-to-severe personal burnout (49.3%) [28].

### Ethical climate: nurses and physicians

Our findings demonstrating that physicians perceived the ethical climate more positive than nurses compares with other studies assessing the perception of the ethical climate for decision-making between physicians and nurses. For example, Jensen and colleagues used the EDMCQ tool in 68 adult ICUs in European countries and the United States and showed that physicians perceived the ethical decision-making climate more positively than nurses [15]. Donkers and colleagues used the EDMCQ tool in ICUs in the Netherlands and showed that intensivists had higher overall climate scores compared with nurses and allied health staff; 4.11 (0.38) 3.90 (0.54) 3.89 (0.43), respectively [3]. In addition, Hamric and Blackhall found that nurses reported a more negative ethical climate than did attending physicians [13].

Regarding the individual climate factors, we showed significant differences between nurses and physicians on all climate factor scores except for “practice and culture of open interdisciplinary reflection” and “active involvement of nurses in end-of-life care and decision making." In contrast, Jensen and colleagues demonstrated significant variations between nurses and physicians for *all* seven climate factors. Finally, Donkers and colleagues confirmed significant differences between nurses and physicians for all factors except for “practice and culture of ethical awareness and support." It appears in general, physicians tend to perceive the ethical climate greater than nurses on a greater number of the climate factors.

### Ethical climate: different ICU types

We showed that the Neonatal/Pediatric ICUs perceived the ethical climate as more positive than the Medical and Surgical ICUs. The nurses and physicians in the Neonatal/Pediatric ICUs also had significantly lower moral distress levels and greater intention to leave scores than the adult ICUs, further supporting a causal pathway between the ethical climate, moral distress, and the intention to leave.

Inspecting the individual climate factors between the different ICUs suggests an explanation for our results. Specifically, both the Neonatal/Pediatric ICUs and Medical ICUs demonstrated higher scores than the Surgical ICUs for the climate factors “culture of not avoiding end-of-life decision making", and “practice and culture of ethical awareness." The differences in the former climate factor might be due to the Surgical ICUs inherently having two teams comprised of surgeons and intensivists involved in the care of patients. In a prospective ethnographic study in a university hospital, tertiary care center involving adult ICUs, Baggs and colleagues noted that management of end-of-life decision-making varied with multiple and shifting attending responsibilities [29]. They observed that attending physicians-initiated discussions about limitation of treatment at different times in different types of ICUs. In particular, discussion of EOLDM by the attending physicians in the Medical ICUs generally began early in the course of the patient’s admission and included multiple parties; patients, families, and other members of the healthcare team. In contrast, discussions about limitation of treatment for surgical patients generally took place later in a patient’s illness trajectory. One participant remarked that “he believed surgeons’ sense of responsibility for the outcome after surgery made it more difficult for them to deal with EOL issues.” Other studies have shown that surgeons compared with intensivists are less reluctant to withdraw postoperative life support, leading to avoidance of end-of-life EOL decisions [30, 31]. These findings regarding the Medical ICUs from the other studies could apply to the Neonatal/Pediatric ICUs as no significant differences were found in this climate factor in our study between these ICU types.

Regarding the differences in the climate factor “practice and culture of ethical awareness", the Neonatal/Pediatric ICUs and Medical ICUs might have encouraged discussions among different members of the staff involving “moral problems” and hence tolerate “different opinion and values concerning end-of-life” (two items included in this climate factor—see Additional file 1). This premise is plausible as in addition to nurses’ presence at goal-of-care family meetings, Baggs and colleagues also noted that for medical patients, “nurses felt empowered to ask questions on rounds, about ‘‘ultimate goals of treatment’’ or about prognosis. In contrast, nurses in the Surgical ICUs most commonly went to a nurse practitioner or care coordinator to discuss end-of-life decision making [29].

The Neonatal/Pediatric ICUs also demonstrated higher ethical climate factor scores on “culture of mutual respect within the interdisciplinary team” and “active involvement of nurses in end-of-life care and decision making compared with the Surgical ICUs. The above noted variations in the culture between these ICU types could also account for the differences in these climate factors.

Although the Neonatal/Pediatric ICUs demonstrated a higher overall climate score than the Medical ICUs, only the climate factor involving “active decision-making by physicians" was higher for the Neonatal/Pediatric ICUs, but this difference was only significant at the *p* = 0.07 level.

### Significance of interprofessional collaboration

The importance of achieving interprofessional collaborative spaces lies in its importance with reducing moral distress and is aligned with recent research suggesting that the intensity of moral distress relates with the ethical climate of the organization [20, 32]. Five of the seven individual climate factors on the EDMCQ represent pivotal elements for accomplishing interprofessional collaboration regarding decision-making at the end-of-life in the ICUs. These factors include: F1: “self-reflective and empowering leadership by physicians”; F2: “open interdisciplinary reflection”; F4: “culture of mutual respect within the interdisciplinary team”; F5: “active involvement of nurses in end-of-life care and decision making"; and F7: “practice and culture of ethical awareness,” Compared with physicians, nurses held a lower perception on the first three of these ethical climate factors. Nurses also demonstrated higher levels on the moral distress factor regarding “suboptimal quality of care due to poor team communication or lack of professionalism", which also questions the extent and quality of collaborative efforts between the nurses and physicians who participated in our survey. In contrast, nurses and physicians ranked highly and similarly the climate factor “active involvement of nurses in end-of-life care and decision making”. This finding contrasts with other studies that demonstrated a discordance between physicians and nurses regarding a role for nurses in decision-making [33–36].

Regarding the distinct ethical climate types, the “good” climate excelled in four of the factors representing interprofessional collaboration (factors F1, F2, F4, and F7). The “poor” climate was deficient in all of the of the interprofessional collaboration factors. It is noteworthy to point out that more than a quarter of the clinicians perceived their ethical climate as “poor”, whereas only approximately 10% of those in the Neonatal/Pediatric ICUs rated their climate as “poor”.

Other studies using the EDMCQ showed that interdisciplinary collaboration does not often occur [3, 15]. Failure to achieve pronounced Interprofessional collaboration can have significant untoward downstream effects. Deficient interdisciplinary communications and teamwork might lead to conflicts and mistakes in inpatient care [37]. Patients and families may also find difficulties finding common ground within the clinical team, negatively influencing trust [15].

Studies have shown a connection between the extent of interprofessional collaboration and an intention to leave. Druwe and colleagues found that interprofessional collaboration, teamwork, and regular interdisciplinary debriefing were associated with a lower risk of intention to leave the job [38]. In a study involving nurses in ICUs in Italy, Karanikola and colleagues demonstrated poor nurse-physician collaboration that appeared to be a pivotal factor in nurses' moral distress and associated with the intention to resign. These authors suggest that enhancing nurse-physician collaboration and participation in end-of-life decisions might alleviate nurses’ moral distress and lessen their intention to leave [8].

Ethical climates that enhance exemplary interprofessional collaboration can have several positive effects. First, it can increase goals of care discussions, decrease ICU length of stay, and improve goal-oriented end-of-life care [12, 39]. It can also lessen moral distress, burnout, and the intention to leave. Van den Bulke and colleagues showed “a protective effect of the ethical climate and intention to leave” among clinicians in 68 adult ICUs in European countries and the United States [12].

### The presence of inappropriate treatments at the end-of-life

Ethical dilemmas at the end-of-life are increasingly common and complex, representing a substantial decision-making challenge for the professional staff in ICUs [2, 40, 41].

Achieving Interprofessional collaboration is pivotal not only to attempt to reach consensus regarding the appropriateness of end-of-life treatments, but to achieve respectful disagreements when consensus is unachievable, which can promote improved end-of-life experiences for clinicians and for patients and their families.

In contrast, an ethical climate that does not support interdisciplinary collaboration towards the appropriateness of end-of-life care can promote overt conflict between the team members, including argumentative communications, distrust, absence of mutual respect, and even avoidance of EOL decision-making altogether [42, 43].

Ethical dilemmas regarding the administration of perceived futile care frequently lead to clinicians’ moral distress and an intention to leave [5, 44, 45]. Other studies support the downstream effects of conflicts regarding the appropriateness of care at the end-of-life. Druwe and colleagues showed that moral distress and the intention to leave the job were associated with a frequent perception of inappropriate CPR [38]. Hamric and colleagues found that the highest moral distress situations for both nurses and physicians involved situations in which they felt pressured to continue with unwarranted aggressive treatment. Nurses perceived such distressing situations more frequently than physicians [13].

The factor “not avoiding end-of-life decisions” scored the lowest on all ethical climates and nurses perceived this climate factor lower than physicians. Different perspectives regarding end-of-life among nurses and physicians might be influenced by their differences in position, responsibilities, authority, and culture, all of which can lead to different perceptions, attitudes, and actions regarding the appropriateness of end-of-life treatment [13]. For example, nurses who are confronted continuously by their patients’ suffering, are guided by an "ethics of care," which can lead to distress among those who may not be able to provide the dignified and peaceful death they desire for their patients, especially if the ethical climate does not empower them to have a voice in end-of-life decision making and fails to support interprofessional mutual respect [46]. In contrast, physicians might adopt more of a focus on patient survival as their authority to withdraw treatment followed by the death of their patients might harbor subsequent guilt. Accordingly, difficulties with acquiescing to the impending deaths of their patients may lead to delays in end-of-life decisions. Additionally, physicians’ personal characteristics might also influence their practice in the withdrawal of life support [47].

### Modifiable elements of the ethical climate

The identified ethical climate factors in the EDMCQ tool represent primary target areas of modification that can enhance interdisciplinary collaboration. Such changes can improve the appropriateness of end-of-life care, lessen moral distress levels, and reduce the intention to leave. Interventions should be designed to enhance mutual respect within the interdisciplinary team and augment the culture and practice of ethical awareness. Reviewing the individual question items in the latter climate factor (see Additional file 1) endorses a focus towards promoting discussions of moral issues involving patients and sharing interdisciplinary opinions and values regarding end-of-life care.

Another improvement target area includes enhancing the process of the end-of-life decision-making. In our study, there were low scores for "not avoiding EOL decision-making." Enhancing this climate factor would involve ensuring that all healthcare team members have the opportunity to attend goal of care discussions and have a voice in the decision-making process. An Inadequate ethical inquiry among members of the ICU team has been associated with excessive interventions in adult intensive care [2, 48]. Diminishing disagreements between clinicians and patients/ families about the appropriateness of treatment may increase satisfaction and trust in the ICU team [37, 49]. Finally, efforts should be directed towards improving clinical training regarding the timely initiation of end-of-life discussions with seriously ill patients and their families [50].

A further modifiable factor involves physicians taking the initiative to improve their decision-making in the ICU. Our study demonstrated a discordance between nurses and physicians regarding the perception of decision-making at the end-of-life in the ICU. Specifically, while physicians rated highly “not avoiding EOL decision making," “empowering others to make decisions," and “active decision making by physicians," nurses, however, shared contrary perceptions on these climate factors.

Finally, one can uncover potential adjustable elements by examining the specific climate factors of the distinct ethical climate types. For example, less than 20% of the clinicians perceived their climate to be “good”. Two factors in this climate that showed the most significant discordance from the other climates included “open interdisciplinary reflection” and "mutual interdisciplinary respect". Interventions should focus on the individual items of these climate factors (see Additional file 1).

### Limitations

We recognize several limitations of this study. First, recruiting a convenience sample might have led to a sample that was not representative of the study population. The presence of self-selection bias in our data cannot be discounted, as only those interested in the topic would be willing to participate. However, our offer of an incentive might have recruited individual who were not necessarily interested in the study topic as well as those thinking they might not have the time [51].

Second, we combined nurse practitioners and bedside nurses within the same group, each of whom might hold different perceptions due to their various roles and interactions with physicians. Finally, our study findings might not be generalizable to other types of institutions, e.g., small community hospitals. We recommend that each institution use the ICU survey tools to determine the ethical climate factors that differ between their professional staff.

## Conclusions

The usefulness of the EDMCQ tool lies in its ability to measure factors contributing to deficiencies in the ethical climate. A focus on these climate factor can guide interventions to enhance the ethical climate for decision making. The EDMCQ can also determine which factors are perceived differently within the various ICU types. The MMD-HP results can also inform targets' area of improvement. For example, the factors showing the most significant discordance between nurses and physicians regarded “ethically inappropriate care due to suboptimal clinical decision making” and “suboptimal quality of care due to poor team communication or lack of professionalism”. Finally, the Neonatal/Pediatric ICUs distinguished itself from the Adult ICUs by displacing lower levels of moral distress for the factor “suboptimal patient care due to organizational restrictions and burden.” These results can help hospital leadership target areas that enhance interdisciplinary collaboration for decision-making, leading to decreased turnover and improved patient care. As hospital systems will vary in their ethical climates for decision-making, the EDMCQ and MMD-HP tools should be employed individually to guide specific interventions for each hospital system.

## Supplementary Information

**Additional file 1.** Question items on each climate construct.

**Additional file 2.** Cluster Analysis Revealing Different Ethical Climates.

**Additional file 3.** Mean Scores and Reliability Results of the Seven Climate Factors.

**Additional file 4.** Identified factors and factor loadings for each item of the Measure of Moral Distress–Healthcare Professionals (MMD-HP) questionnaire.

**Additional file 5.** Factors Identified for the MMD-HP from factor analysis.

**Additional file 6.** Test for linearity.

**Additional file 7.** Mean Climate Factor Scores of the Distinct Ethical Climates.

## Data Availability

The datasets used and/or analyzed during the current study are available from https://osf.io/d3w2b/?view_only=fed03662a0594beca1977d1a0ea3f073

## References

[1] Van den Bulcke B, Piers R, Jensen HI, Malmgren J, Metaxa V, Reyners AK (2018). Ethical decision-making climate in the ICU: theoretical framework and validation of a self-assessment tool. BMJ Qual Saf.

[2] Benoit DD, Jensen HI, Malmgren J, Metaxa V, Reyners AK, Darmon M (2018). Outcome in patients perceived as receiving excessive care across different ethical climates: a prospective study in 68 intensive care units in Europe and the USA. Intensive Care Med.

[3] Donkers MA, Gilissen V, Candel M, van Dijk NM, Kling H, Heijnen-Panis R (2021). Moral distress and ethical climate in intensive care medicine during COVID-19: a nationwide study. BMC Med Ethics.

[4] Jameton A (1984). Nursing Practice: the ethical issues.

[5] Dzeng E, Colaianni A, Roland M, Levine D, Kelly MP, Barclay S (2016). Moral Distress Amongst American Physician Trainees Regarding Futile Treatments at the End of Life: A Qualitative Study. J Gen Intern Med.

[6] Houston S, Casanova MA, Leveille M, Schmidt KL, Barnes SA, Trungale KR (2013). The intensity and frequency of moral distress among different healthcare disciplines. J Clin Ethics.

[7] Epstein EG, Whitehead PB, Prompahakul C, Thacker LR, Hamric AB (2019). Enhancing Understanding of Moral Distress: The Measure of Moral Distress for Health Care Professionals. AJOB Empir Bioeth.

[8] Karanikola MN, Albarran JW, Drigo E, Giannakopoulou M, Kalafati M, Mpouzika M et al. Moral distress, autonomy and nurse-physician collaboration among intensive care unit nurses in Italy. J Nurs Manag. 2014;22(4):472–84. doi: https:// doi. org/ 10. 1111/ jonm. 12046. 10.1111/jonm.12046 23489299

[9] Ganz FD, Raanan O, Khalaila R, Bennaroch K, Scherman S, Bruttin M (2013). Moral distress and structural empowerment among a national sample of Israeli intensive care nurses. J Adv Nurs.

[10] Ozden D, Karagozoglu S, Yildirim G. Intensive care nurses' perception of futility: job satisfaction and burnout dimensions. Nurs Ethics. 2013;20(4):436–47. doi: https:// doi. org/ 10. 1177/ 09697 33012 466002. 10.1177/0969733012466002 23411368

[11] Varcoe C, Pauly B, Storch J, Newton L, Makaroff K. Nurses' perceptions of and responses to morally distressing situations. Nurs Ethics. 2012;19(4):488–500. doi:. https:// doi. org/ 10. 1177/ 09697 33011 436025. 10.1177/0969733011436025 22619236

[12] Van den Bulcke B, Metaxa V, Reyners AK, Rusinova K, Jensen HI, Malmgren J (2020). Ethical climate and intention to leave among critical care clinicians: an observational study in 68 intensive care units across Europe and the United States. Intensive Care Med.

[13] Hamric AB, Blackhall LJ (2007). Nurse-physician perspectives on the care of dying patients in intensive care units: collaboration, moral distress, and ethical climate. Crit Care Med.

[14] ALEX MANN, CHRISTINE CONDON, STEVE EARLEY. 2021 COVID metrics: What the data shows us about the pandemic in Maryland last year. Baltimore Sun. January 13, 2022 Accessibe at: https://www.baltimoresun.com/coronavirus/bs-md-covid-2021-numbers-20220113-foepv44n5zejphafvwnp3zphoe-story.html.

[15] Jensen HI, Hebsgaard S, Hansen TCB, Johnsen RFA, Hartog CS, Soultati I et al. Perceptions of Ethical Decision-Making Climate Among Clinicians Working in European and U.S. ICUs: Differences Between Nurses and Physicians. Crit Care Med. 2019;47(12):1716–23. doi:10.1097/CCM.0000000000004017. 10.1097/CCM.0000000000004017 31625980

[16] Dzeng E, Curtis JR (2018). Understanding ethical climate, moral distress, and burnout: a novel tool and a conceptual framework. BMJ Qual Saf.

[17] Azoulay E, Cariou A, Bruneel F, Demoule A, Kouatchet A, Reuter D et al. Symptoms of Anxiety, Depression, and Peritraumatic Dissociation in Critical Care Clinicians Managing Patients with COVID-19. A Cross-Sectional Study. Am J Respir Crit Care Med. 2020;202(10):1388–98. doi:10.1164/rccm.202006-2568OC. 10.1164/rccm.202006-2568OC PMC7667906 32866409

[18] Kok N, van Gurp J, Teerenstra S, van der Hoeven H, Fuchs M, Hoedemaekers C (2021). Coronavirus Disease 2019 Immediately Increases Burnout Symptoms in ICU Professionals: A Longitudinal Cohort Study. Crit Care Med.

[19] Hwang JI, Park HA (2014). Nurses' perception of ethical climate, medical error experience and intent-to-leave. Nurs Ethics.

[20] Altaker KW, Howie-Esquivel J, Cataldo JK (2018). Relationships Among Palliative Care, Ethical Climate, Empowerment, and Moral Distress in Intensive Care Unit Nurses. Am J Crit Care.

[21] Whitford B, Nadel AL, Fish JD (2018). Burnout in pediatric hematology/oncology-time to address the elephant by name. Pediatr Blood Cancer.

[22] Trotochaud K, Coleman JR, Krawiecki N, McCracken C (2015). Moral Distress in Pediatric Healthcare Providers. J Pediatr Nurs.

[23] Dyrbye LN, Shanafelt TD, Sinsky CA (2017). Burnout among health care professionals: a call to explore and address this underrecognized threat to safe, high-quality care. NAM Perspectives.

[24] Sauerland J, Marotta K, Peinemann MA, Berndt A, Robichaux C (2015). Assessing and addressing moral distress and ethical climate Part II: neonatal and pediatric perspectives. Dimens Crit Care Nurs.

[25] Atabay G, Cangarli BG, Penbek S (2015). Impact of ethical climate on moral distress revisited: multidimensional view. Nurs Ethics.

[26] Fumis RRL, Junqueira Amarante GA, de Fatima NA, Vieira Junior JM (2017). Moral distress and its contribution to the development of burnout syndrome among critical care providers. Ann Intensive Care.

[27] Silverman HJ, Kheirbek RE, Moscou-Jackson G, Day J. Moral distress in nurses caring for patients with Covid-19. Nurs Ethics. 2021:9697330211003217. doi:10.1177/09697330211003217. 10.1177/09697330211003217 33910406

[28] Chor WPD, Ng WM, Cheng L, Situ W, Chong JW, Ng LYA (2021). Burnout amongst emergency healthcare workers during the COVID-19 pandemic: A multi-center study. Am J Emerg Med.

[29] Baggs JG, Schmitt MH, Prendergast TJ, Norton SA, Sellers CR, Quinn JR (2012). Who is attending? End-of-life decision making in the intensive care unit. J Palliat Med.

[30] Paul Olson TJ, Brasel KJ, Redmann AJ, Alexander GC, Schwarze ML (2013). Surgeon-reported conflict with intensivists about postoperative goals of care. JAMA Surg.

[31] Schwarze ML, Redmann AJ, Alexander GC, Brasel KJ (2013). Surgeons expect patients to buy-in to postoperative life support preoperatively: results of a national survey. Crit Care Med.

[32] Pauly B, Varcoe C, Storch J, Newton L (2009). Registered nurses' perceptions of moral distress and ethical climate. Nurs Ethics.

[33] Ferrand E, Lemaire F, Regnier B, Kuteifan K, Badet M, Asfar P (2003). Discrepancies between perceptions by physicians and nursing staff of intensive care unit end-of-life decisions. Am J Respir Crit Care Med.

[34] Benbenishty J, Ganz FD, Lippert A, Bulow HH, Wennberg E, Henderson B (2006). Nurse involvement in end-of-life decision making: the ETHICUS Study. Intensive Care Med.

[35] Jensen HI, Ammentorp J, Erlandsen M, Ording H (2011). Withholding or withdrawing therapy in intensive care units: an analysis of collaboration among healthcare professionals. Intensive Care Med.

[36] Thomas EJ, Sexton JB, Helmreich RL (2003). Discrepant attitudes about teamwork among critical care nurses and physicians. Crit Care Med.

[37] Donovan AL, Aldrich JM, Gross AK, Barchas DM, Thornton KC, Schell-Chaple HM (2018). Interprofessional Care and Teamwork in the ICU. Crit Care Med.

[38] Druwe P, Monsieurs KG, Gagg J, Nakahara S, Cocchi MN, Elo G (2021). Impact of perceived inappropiate cardiopulmonary resuscitation on emergency clinicians' intention to leave the job: Results from a cross-sectional survey in 288 centres across 24 countries. Resuscitation.

[39] Wocial L, Ackerman V, Leland B, Benneyworth B, Patel V, Tong Y (2017). Pediatric Ethics and Communication Excellence (PEACE) Rounds: Decreasing Moral Distress and Patient Length of Stay in the PICU. HEC Forum.

[40] Piers RD, Azoulay E, Ricou B, Dekeyser Ganz F, Decruyenaere J, Max A (2011). Perceptions of appropriateness of care among European and Israeli intensive care unit nurses and physicians. JAMA.

[41] Piers RD, Azoulay E, Ricou B, DeKeyser GF, Max A, Michalsen A (2014). Inappropriate care in European ICUs: confronting views from nurses and junior and senior physicians. Chest.

[42] Azoulay E, Timsit JF, Sprung CL, Soares M, Rusinova K, Lafabrie A (2009). Prevalence and factors of intensive care unit conflicts: the conflicus study. Am J Respir Crit Care Med.

[43] Michalsen A, Long AC, DeKeyser GF, White DB, Jensen HI, Metaxa V (2019). Interprofessional Shared Decision-Making in the ICU: A Systematic Review and Recommendations From an Expert Panel. Crit Care Med.

[44] Mobley MJ, Rady MY, Verheijde JL, Patel B, Larson JS (2007). The relationship between moral distress and perception of futile care in the critical care unit. Intensive Crit Care Nurs.

[45] Schwarzkopf D, Ruddel H, Thomas-Ruddel DO, Felfe J, Poidinger B, Matthaus-Kramer CT (2017). Perceived Nonbeneficial Treatment of Patients, Burnout, and Intention to Leave the Job Among ICU Nurses and Junior and Senior Physicians. Crit Care Med.

[46] Epstein EG (2008). End-of-life experiences of nurses and physicians in the newborn intensive care unit. J Perinatol.

[47] Christakis NA, Asch DA (1995). Physician characteristics associated with decisions to withdraw life support. Am J Pub Health.

[48] Dzeng E, Colaianni A, Roland M, Chander G, Smith TJ, Kelly MP (2015). Influence of institutional culture and policies on do-not-resuscitate decision making at the end of life. JAMA Intern Med.

[49] Wilson ME, Dobler CC, Zubek L, Gajic O, Talmor D, Curtis JR (2019). Prevalence of Disagreement About Appropriateness of Treatment Between ICU Patients/Surrogates and Clinicians. Chest.

[50] Sutherland R (2019). Dying Well-Informed: The Need for Better Clinical Education Surrounding Facilitating End-of-Life Conversations. Yale J Biol Med.

[51] Patrick ME, Singer E, Boyd CJ, Cranford JA, McCabe SE (2013). Incentives for college student participation in web-based substance use surveys. Addict Behav.

