# Supplementary material for: Ethical decision-making climate, moral distress, and intention to leave among ICU professionals in a tertiary academic hospital center
Source: BMC Med Ethics. 2022 Apr 19;23:45. doi: 10.1186/s12910-022-00775-y (PMC9017406; doi:10.1186/s12910-022-00775-y)
Supplement: Supplementary file 1 — Additional file 1. Question items on each climate construct. [file 12910_2022_775_MOESM1_ESM.docx]

**Additional File 1**

**Questions on each climate construct (from Van den Bulcke et. al. [5])**

**F1: Self-reflective and empowering leadership by physicians**

L-5 Physicians in charge help team members settle their differences.

L-6 Physicians in charge trust the team members to exercise good judgement.

L-7 Physicians in charge permit the team members to use their own judgement in solving problems.

L-8 Physicians in charge encourage initiative in the team members.

L-9 Physicians in charge treat all team members as their equals.

L-11 Physicians in charge are well aware of their own emotions and attitudes.

L-13 Physicians in charge dare to show their vulnerability.

**F2: Practice and culture of open interdisciplinary reflection**

I-1 There are regular opportunities for open informal dialogue between healthcare providers.

I-2 There is regular structured and formal dialogue between the various disciplines within the team to discuss patient care.

I-3 We regularly reflect on the quality of care provided from the various points of view of the staff.

I-4 The teams are well coordinated/managed.

I-5 There is an open and constructive culture in the department such that criticism can be easily expressed.

I-6 Discussions about patients lead to greater understanding and agreements.

I-11 The culture in my ICU makes it easy to learn from the errors of others.

**F3: Culture of not avoiding EOL decisions**

E-8† Death is not perceived as a treatment failure, so decisions to withdraw or withhold therapy are seldom postponed.

E-9† EOL decisions are not frequently postponed.

E-10† Patients with little chance of recovery are not frequently admitted.

E-11† Patients with little chance of recovery do not frequently occupy an ICU bed which other patients would benefit more from.

**F4: Culture of mutual respect within the interdisciplinary team**

I-7 I am always regarded and addressed by everyone in the team as a full-fledged team member.

I-8 Team members from another discipline respect my work.

I-9 I have confidence in the professional competence of my team members.

**F5: Active involvement of nurses in EOL care and decision-making (DM)**

E-5 Nurses are present during the communication of end-of-life information to the family.

E-6 Nurses are involved in end-of-life decision-making.

E-7 Nurses and physicians collaborate well with one another during end-of-life situations.

**F6: Active decision-making by physicians**

L-2 Physicians in charge make accurate and timely decisions.

L-3 Physicians in charge take full charge when emergencies arise.

L-4† Physicians in charge are not hesitant about taking initiative in the group.

L-12 Physicians in charge are well aware of their role model function.

**F7: Practice and culture of ethical awareness**

E-1 My colleagues understand my thoughts/feelings about difficult end-of-life decisions.

E-2 Different opinions and values concerning end of life are tolerated.

E-3 We talk about moral problems.

E-4 There is a structured, formal debriefing after difficult patient care situation.
