# Supplementary material for: Ethical decision-making climate, moral distress, and intention to leave among ICU professionals in a tertiary academic hospital center
Source: BMC Med Ethics. 2022 Apr 19;23:45. doi: 10.1186/s12910-022-00775-y (PMC9017406; doi:10.1186/s12910-022-00775-y)
Supplement: Supplementary file 2 — Additional file 2. Cluster Analysis Revealing Different Ethical Climates. [file 12910_2022_775_MOESM2_ESM.docx]

**Additional File 2**


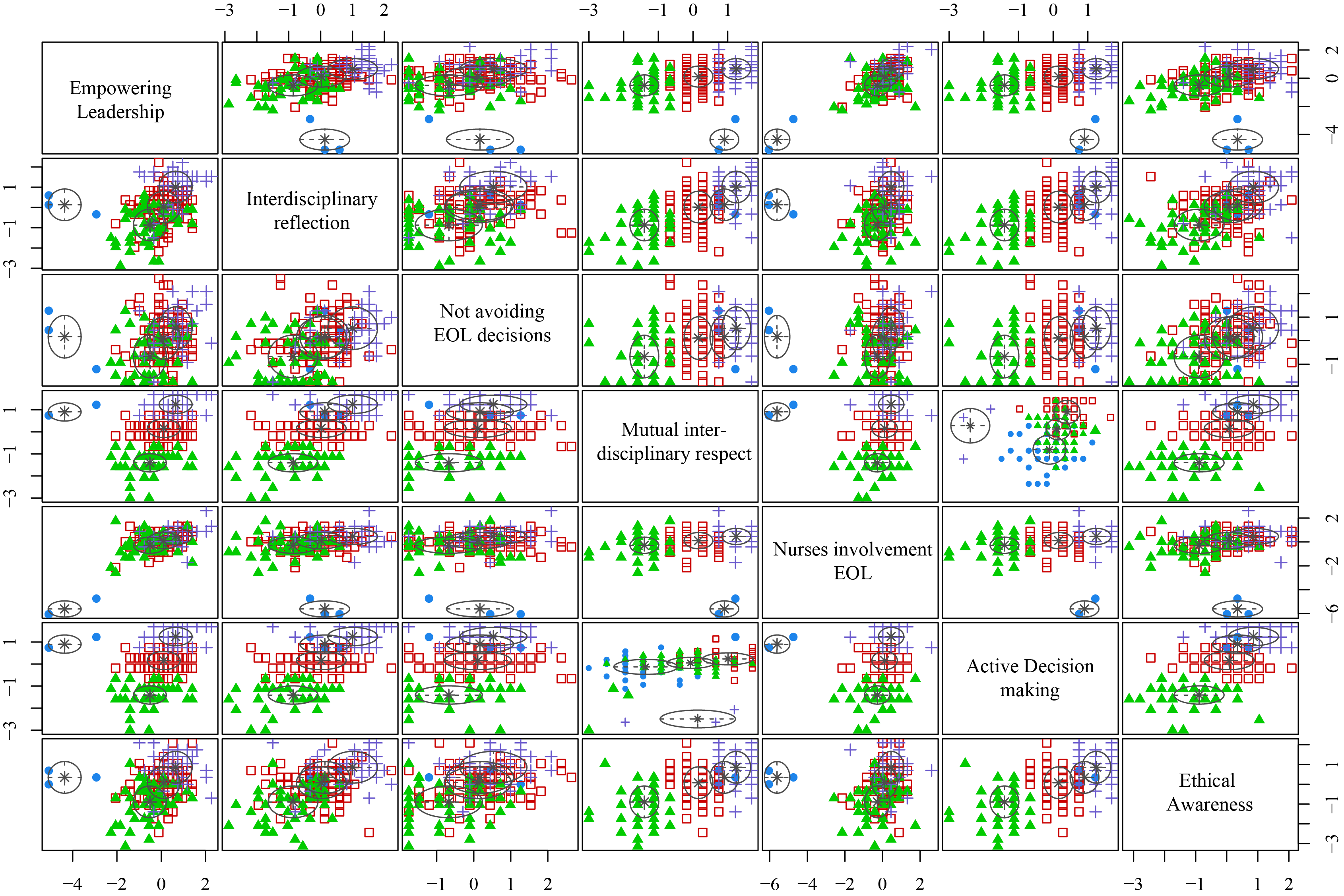


**Figure 1: Identification of ethical climates: Cluster Analysis**

We found scores to cluster within four mutually exclusive ethical climates: cluster 1 (purple + signs) has high scores representing “good” climate, cluster 4 (blue dots) with low scores representing “poor” climate, cluster 3 (red squares) with average scores representing “average” climate (+) and cluster 2 (green triangles) with lower scores for several of the climate factors compared with “average (+) and hence, it is denoted by “average (-).
