# Supplementary material for: Ethical decision-making climate, moral distress, and intention to leave among ICU professionals in a tertiary academic hospital center
Source: BMC Med Ethics. 2022 Apr 19;23:45. doi: 10.1186/s12910-022-00775-y (PMC9017406; doi:10.1186/s12910-022-00775-y)
Supplement: Supplementary file 3 — Additional file 3. Mean Scores and Reliability Results of the Seven Climate Factors. [file 12910_2022_775_MOESM3_ESM.docx]

**Additional File 3**

Mean scores (SD) and internal consistency of the seven climates factors of the EDMCQ.

| **Factor no.** | **Factor description** | **Number of items per factor** | **Mean score**  **(SD)** | **Cronbach α^a^** |
| --- | --- | --- | --- | --- |
| 1 | Self-reflective and empowering leadership by physicians | 7 | 3.36 (1.0) | 0.78 |
| 2 | Practice and culture of open interdisciplinary reflection | 7 | 3.63 (0.95) | 0.86 |
| 3 | Culture of not avoiding EOL decisions | 4 | 2.60 (0.9) | 0.87 |
| 4 | Culture of mutual respect within the interdisciplinary team | 3 | 3.8 (1.1) | 0.84 |
| 5 | Active involvement of nurses in EOL care and decision-making (DM) | 3 | 3.49 (1.3) | 0.80 |
| 6 | Active decision-making by physicians | 4 | 3.30 (0.8) | 0.82 |
| 7 | Practice and culture of ethical awareness | 4 | 3.5 (0.88) | 0.79 |
