# Supplementary material for: Ethical decision-making climate, moral distress, and intention to leave among ICU professionals in a tertiary academic hospital center
Source: BMC Med Ethics. 2022 Apr 19;23:45. doi: 10.1186/s12910-022-00775-y (PMC9017406; doi:10.1186/s12910-022-00775-y)
Supplement: Supplementary file 4 — Additional file 4. Identified factors and factor loadings for each item of the Measure of Moral Distress–Healthcare Professionals (MMD-HP) questionnaire. [file 12910_2022_775_MOESM4_ESM.docx]

| **Additional File 4:** Identified factors and factor loadings for each item of the Measure of Moral Distress –Healthcare Professionals (MMD-HP) questionnaire* | |
| --- | --- |
|  | |
| **Items per factor** | **Factor loading**** |
| **Factor 1. Ethically Inappropriate Care Due to Suboptimal Clinical Decision Making** |  |
| 5. Continue to provide aggressive treatment for a person who is most likely to die regardless of this treatment when no one will make a decision to withdraw it. | 0.77 |
| 1. Witness healthcare providers giving “false hope” to a patient or family. | 0.76 |
| 2. Follow the family’s insistence to continue aggressive treatment even though I believe it is not in the best interest of the patient. | 0.72 |
| 8. Participate in care that causes unnecessary suffering or does not adequately relieve pain or symptoms. | 0.67 |
| 3. Feel pressured to order or carry out orders for what I consider to be unnecessary or inappropriate tests and treatments. | 0.61 |
| 24. Been required to care for patients who have unclear or inconsistent treatment plans or who lack goals of care. | 0.53 |
| **Factor 2. Suboptimal patient care due to organizational restrictions/ burden** |  |
| 17. Experience compromised patient care due to lack of resources/bed capacity. | 0.61 |
| 18. Experience lack of administrative action or support for a problem that is compromising patient care. | 0.6 |
| 16. Been required to care for more patients than I can safely care for. | 0.54 |
| 23. Feel required to overemphasize tasks and productivity or quality measures at the expense of patient care. | 0.52 |
| 19. Have excessive documentation requirements that compromise patient care. | 0.48 |
| 13. Been required to work with other healthcare team members who are not as competent as patient care requires. | 0.43 |
| 7. Been required to care for patients whom I do not feel qualified to care for. | 0.42 |
| 4. Been unable to provide optimal care due to pressures from administrators or insurers to reduce costs. | 0.40 |
| 9. Watch patient care suffer because of a lack of provider continuity. | 0.33 |
| **Factor 3. Suboptimal quality of care due to poor team communication or lack of professionalism** |  |
| 27. Work with team members who do not treat vulnerable or marginalized patients with dignity and respect. | 0.59 |
| 26. Participate on a team that gives inconsistent messages to a patient/family | 0.52 |
| 14. Witness low quality of patient care due to poor team communication. | 0.51 |
| **Factor 4. Culture of fear and power hierarchy** |  |
| 21 Feel unsafe/bullied amongst my own colleagues. | 0.70 |
| 20. Fear retribution if I speak up. | 0.69 |
| 12. Participate in care that I do not agree with but do so because of fears of litigation. | 0.55 |
| 11. Witness a violation of a standard of practice or a code of ethics and not feel sufficiently supported to report the violation. | 0.53 |
| 25. Work within power hierarchies in teams, units, and my institution that compromise patient care. | 0.34 |

* The numbers for each item represent the numbering of the original MMD-HP

**The relationship of each variable to the underlying factor is expressed by the factor loading.
