# Supplementary material for: Ethical decision-making climate, moral distress, and intention to leave among ICU professionals in a tertiary academic hospital center
Source: BMC Med Ethics. 2022 Apr 19;23:45. doi: 10.1186/s12910-022-00775-y (PMC9017406; doi:10.1186/s12910-022-00775-y)
Supplement: Supplementary file 5 — Additional file 5. Factors Identified for the MMD-HP from factor analysis. [file 12910_2022_775_MOESM5_ESM.docx]

**Additional File 5**

MMD-HP factors identified, number of items, percentage of variance, mean scale score and Cronbach’s alpha explained by each factor (n = 206)

| **Factor no.** | **Factor description** | **Percentage of variance explained by factor** | **Number of items per factor** | **Mean scale score** | **Cronbach α^a^** |
| --- | --- | --- | --- | --- | --- |
| 1 | Ethically Inappropriate Care Due to Suboptimal Clinical Decision Making | 39.4 | 6 | 7.07 | 0.85 |
| 2 | Suboptimal patient care due to organizational restrictions/ burden | 12.1 | 9 | 4.14 | 0.81 |
| 3 | Suboptimal quality of care due to poor team communication or lack of professionalism | 4.8 | 3 | 4.11 | 0.77 |
| 4 | Culture of fear and power hierarchy | 3.2 | 5 | 2.23 | 0.80 |

^a^Cronbach’s alpha ranges from 0 to 1: the higher the coefficient, the more consistent the scale.
