# Supplementary material for: Ethical decision-making climate, moral distress, and intention to leave among ICU professionals in a tertiary academic hospital center
Source: BMC Med Ethics. 2022 Apr 19;23:45. doi: 10.1186/s12910-022-00775-y (PMC9017406; doi:10.1186/s12910-022-00775-y)
Supplement: Supplementary file 6 — Additional file 6. Test for linearity. [file 12910_2022_775_MOESM6_ESM.docx]

**Additional File 6**

**Test for linearity**

Pearson’s correlation coefficient was computed to identify statistically significant associations between moral distress and ethical climate, moral distress, and intent to leave, and ethical climate and intent to leave. Pearson’s correlation coefficient assumes linearity which was tested by inspecting the Residuals and Fitted plot.

**Panel A** **Panel B**


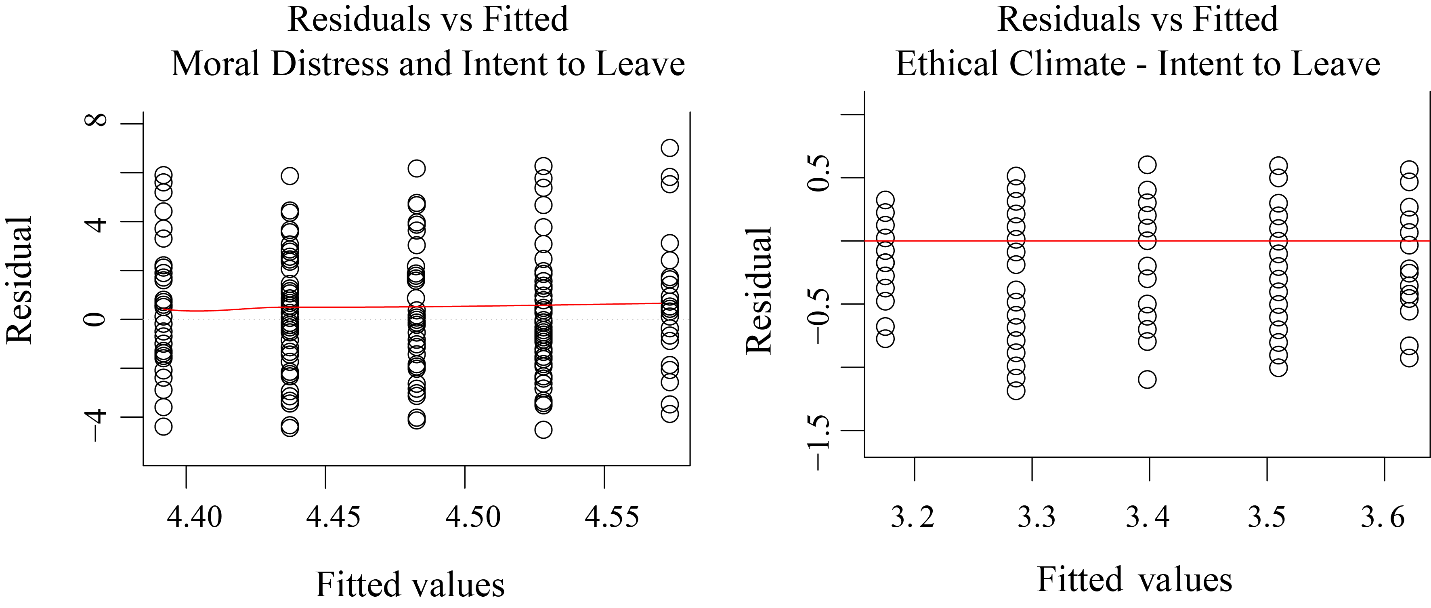


The plot of the residuals lacks any fitted pattern indicating linearity in the data.

**Games-Howell post-hoc test**

The Games Howell post-hoc test is used when the assumption of homogeneity of variances is violated. We used Levene’s test for measure of homogeneity of variances and found a significance value of <0.001 for all our tested variables showing that the homogeneity of variance cannot be assumed and this Games-Howell is an appropriate post-hoc test to use.
