# Supplementary material for: Ethical decision-making climate, moral distress, and intention to leave among ICU professionals in a tertiary academic hospital center
Source: BMC Med Ethics. 2022 Apr 19;23:45. doi: 10.1186/s12910-022-00775-y (PMC9017406; doi:10.1186/s12910-022-00775-y)
Supplement: Supplementary file 7 — Additional file 7. Mean Climate Factor Scores of the Distinct Ethical Climates. [file 12910_2022_775_MOESM7_ESM.docx]

**Additional File 7: Mean Climate Factor Scores of the Distinct Ethical Climates**

|  | F1 Empowering Leadership | F2 Open Interdisciplinary Reflection | F3 Not avoiding EOL decisions | F4 Mutual Interdisciplinary Respect | F5 Nurses involvement in EOL DM | F6 Active DM by physicians | F7 Practice and Culture of Ethical Awareness |
| --- | --- | --- | --- | --- | --- | --- | --- |
| Good | 3.86 | 4.38 | 3.11 | 4.70 | 3.77 | 4.20 | 4.15 |
| Average (+) | 3.56 | 3.77 | 3.39 | 3.93 | 3.59 | 3.43 | 3.75 |
| Average (-) | 3.21 | 3.59 | 2.15 | 3.97 | 3.39 | 3.47 | 3.49 |
| Poor | 2.50 | 3.05 | 1.94 | 2.89 | 2.39 | 2.95 | 2.83 |
